# Role of TMPRSS2-ERG Gene Fusion in Negative Regulation of PSMA Expression

**DOI:** 10.1371/journal.pone.0021319

**Published:** 2011-06-24

**Authors:** Lihong Yin, Pravin Rao, Paul Elson, Jianghua Wang, Michael Ittmann, Warren D. W. Heston

**Affiliations:** 1 Department of Cancer Biology, Cleveland Clinic, Cleveland, Ohio, United States of America; 2 Glickman Urological and Kidney Institute, Cleveland Clinic, Cleveland, Ohio, United States of America; 3 Department of Quantitative Health Sciences, Cleveland Clinic, Cleveland, Ohio, United States of America; 4 Department of Pathology and Immunology, Baylor College of Medicine, and the Michael E. DeBakey Veterans Affairs Medical Center (VAMC), Houston, Texas, United States of America; Bauer Research Foundation, United States of America

## Abstract

Prostate specific membrane antigen (PSMA) is overexpressed in prostatic adenocarcinoma (CaP), and its expression is negatively regulated by androgen stimulation. However, it is still unclear which factors are involved in this downregulation. *TMPRSS2-ERG* fusion is the most common known gene rearrangement in prostate carcinoma. Androgen stimulation can increase expression of the *TMPRSS2-ERG* fusion in fusion positive prostate cancer cells. The purpose of this investigation is to determine whether PSMA expression can be regulated by the *TMPRSS2-ERG* gene fusion. We employed two PSMA positive cell lines: VCaP cells, which harbor *TMPRSS2-ERG* fusion, and LNCaP cells, which lack the fusion. After 24 hours of androgen treatment, TMPRSS2-ERG mRNA level was increased in VCaP cells. PSMA mRNA level was dramatically decreased in VCaP cells, while it only has moderate change in LNCaP cells. Treatment with the androgen antagonist flutamide partially restored PSMA expression in androgen-treated VCaP cells. Knocking down ERG by siRNA in VCaP cells enhances PSMA expression both in the presence and absence of synthetic androgen R1881. Overexpressing *TMPRSS2-ERG* fusions in LNCaP cells downregulated PSMA both in the presence or absence of R1881, while overexpressing wild type ERG did not. Using PSMA-based luciferase reporter assays, we found *TMPRSS2-ERG* fusion can inhibit PSMA activity at the transcriptional level. Our data indicated that downregulation of PSMA in androgen-treated VCaP cells appears partially mediated by *TMPRSS2-ERG* gene fusion.

## Introduction

Prostate specific membrane antigen (PSMA) is a type II transmembrane glycoprotein overexpressed in prostate carcinoma. The protein consists of 750 amino acids with a molecular weight ∼100 kDa [1]. The extracellular domain has activities as a folate hydrolase (cleaving the terminal glutamates from γ-linked polyglutamates) and NAALADase (cleaving the terminal glutamate from α-linked N-acetylaspartyl glutamate). PSMA can undergo internalization, and its intracellular domain is known to bind to actin binding protein filamin A [2], [3]. Nearly all prostate cancer cells express PSMA, and its expression has been correlated with aggressive disease [4], [5], [6]. In addition to normal prostate gland, benign prostatic hyperplasia (BPH), and prostate cancer (PCa), PSMA is also expressed in the neovasculature of multiple solid tumors [7], [8], [9], [10]. Higher PSMA expression is also found in cancer cells from castration-resistant prostate cancer patients. Increased PSMA expression is reported to correlate with the risk of early prostate cancer recurrence after radical prostatectomy [5], [11], [12]. Investigating the mechanism of PSMA regulation will allow us to better understand the mechanisms and functions of PSMA in prostate cancer.

A common genetic alteration in prostate cancer is a signature gene fusion between the androgen-regulated *TMPRSS2* and the transformation-specific (ETS) transcription factor family members [13], [14], [15], [16]. Of those, the *TMPRSS2-ERG* fusion is the most common known gene rearrangement in prostate cancer. Studies found that approximately 50% of prostate cancers harbor *TMPRSS2-ERG* fusions, of which greater than 90% over-express ERG [17]. TMPRSS2-ERG fusions alter prostate cancer progression by promoting cell invasion, activating C-MYC oncogene and abrogating prostate epithelial differentiation [15], [18], [19]. Recent publications have reported that prostate cancer containing *TMPRSS2-ERG* fusions are significantly enriched for loss of the tumor suppressor PTEN [20]. *TMPRSS2-ERG* cooperates with loss of PTEN to further promote prostate cancer progression [20], [21]. However, it has been controversial as to whether this fusion product results in increased aggressive behavior in the prostate. While a significant amount of investigations have been done on the tumor biological functions of TMPRSS2 fusion-driven ETS overexpression in prostate cancer, few papers have reported on its potential downstream targets.

While PSMA is upregulated overall in prostate cancer, it is strongly overexpressed in 50% of prostate cancer specimens [5], [6], [22]. Although *TMPRSS2-ERG* is also found in 50% of prostate cancer specimens [14], [15], [17], it is unknown if, or to what extent these populations overlap. PSMA is upregulated by androgen ablation, and androgens can stimulate *TMPRSS2-ERG* gene fusion, as the TMPRSS2 promoter has an androgen-responsive element. This information provides a potential link between inhibition of PSMA by androgen and ERG expression in fusion-positive prostate cancer cells. We hypothesized that PSMA expression could be regulated by the *TMPRSS2-ERG* fusion. VCaP cells express the *TMPRSS2-ERG* fusion, while LNCaP cells do not. Therefore, we investigated PSMA regulation in TMPRSS2-ERG fusion-positive VCaP and fusion-negative LNCaP prostate cancer cells. Our data suggest that downregulation of PSMA by androgen is mediated by TMPRSS2-ERG gene fusion in VCaP cells.

## Materials and Methods

### Cell culture and transfection

Lymph node metastasis-derived LNCaP cells (ATCC) were grown in RPMI 1640 medium (Mediatech), and bone metastasis-derived VCaP cells (ATCC) were grown in modified DMEM medium (Cat# 30-2002, ATCC), both with 10% fetal bovine serum (USB Corp) in a humidified atmosphere of 5% CO_2_ at 37°C. At 60–80% confluence cell lines were incubated with vehicle DMSO or 10 µM flutamide (androgen receptor antagonist; Sigma) for 2 hours before treatment with 5 nM of R1881 (synthetic androgen, Perkin Elmer) for 24 hours. *TMPRSS2-ERG* fusion transcript isoforms (III, III+72, VI, VI+72) [18] or ERG cDNA (RC218892, OriGene Technologies, Inc.) were transfected into LNCaP cells by Lipofectamine 2000 (Cat# 11668-019, Invitrogen). The empty vectors were pcDNA3.1 and pCMV6-Entry respectively. Transfections were performed according to the manufacturer's instruction (Invitrogen). The ratio of DNA (μg) to Lipofectamine 2000 (μl) is 1∶2.5. Expression of ERG fusion proteins and wild type ERG were checked by western blot.

### ERG knockdown with small interfering RNA (siRNA)

For siRNA knockdown of ERG, siRNA against human ERG (NM_004440) was designed as 5′-GACATCCTTCTCTCACAT-3′ (Ambion, Austin, TX) or purchased from Dharmacon (D-001210-01; Lafayette, CO). They are designated as siERG-1 (from Ambion) and siERG-2 (from Dharmacon) respectively. Negative control #1 siRNA (non-targeting) (Cat#4390843; Ambion) or siRNA against ERG was transfected into VCaP cells as indicated, using Oligofectamine (Cat# 12252-011, Invitrogen). Transfections were performed according to the manufacturer's instruction (Invitrogen). VCaP cells were seeded into 12-well plate one day before transfection. 200 nM of siRNA and 3 µl of Oligofectamine were used for each transfection. After 24 hours, a second identical transfection was carried out and cells were harvested 24 hours later. siRNA knockdown of ERG was tested by both quantitative polymerase chain reaction (qPCR) and immunoblot analysis. Experiments were done in triplicate.

### Detection of proteins by Western Blot analysis

Cells were lysed in RIPA buffer (50 mM Tris-HCl, pH 7.5, 150 mM NaCl, 1% Igepal CA-630, 0.5% Na Deoxycholate, 0.1% SDS, 1 mM EDTA, 50 mM NaF, 200 µM Na Orthovanadate) supplemented with protease inhibitor cocktail (Cat# 11836153001, Roche). Protein samples were run in 7.5% Tris-HCL precast ready gel (Cat# 161-1154, BIO-RAD) and transferred into PVDF membrane. Blots were blocked in 5% skim milk and incubated with anti-ERG antibody (1: 1000 dilution) (Cat# sc-354, Santa Cruz Biotechnology), anti-PSMA antibody GCP-04 (1∶10000 dilution) (gift from Dr. Jan Konvalinka, Academy of Sciences of the Czech Republic, Prague, Czech Republic), anti-V5 antibody (1: 1000 dilution) (Cat# R961-25, Invitrogen), or anti-GAPDH (1: 20000 dilution) (Cat# 2-RGM2, Advanced ImmunoChemical Inc.). HRP labeled goat anti-rabbit or goat anti-mouse was used as secondary antibody (1∶5000; Pierce). Signal was detected by chemiluminescent substrate from Pierce (Cat# 34080).

### Real-Time RT-PCR analysis

Gene expression was verified by real-time reverse transcription (RT)-PCR. RNA was isolated using RNA-Bee (Cat# CS-105B, TEL-TEST, INC.) and purified by RNeasy mini kit (Cat# 74104, QIAGEN). The first strand of cDNA was synthesized from RNA samples with an iScript cDNA synthesis kit (Cat# 170-8890, Bio-Rad Laboratories). 500 ng of total RNA from each sample was applied to each reaction. 20 µL of complete reaction mix was incubated at 25 °C for 5 min, followed by 42 °C for 30 min and 85 °C for 5 min. After cDNA synthesis, 2 µL of reaction was then used to set up PCR reactions with an iQ SYBR Green Supermix kit (Cat# 170-8882, Bio-Rad). Total reaction volume for PCR was set to 20 µL in 2x SYBR Green Supermix with gene specific primers. Reactions were run at 95 °C for 10 sec, 60 °C for 30 sec followed by 40 cycles. Fluorescent data were collected in an iCycler iQ detection system (Bio-Rad). For each sample in a given experiment, duplicate reactions were set up with a primer pair for the gene of interest, as well as duplicate reactions with a primer pair for phosphogluconate dehydrogenase (PGD), which was used as an endogenous reference. Using the comparative threshold cycles (CT) method, the quantification normalized to PGD and relative to untreated, parental cells was performed. Fold changes were calculated using the formula 2^−ΔΔCT^, where ΔCt is Ct_(target gene)_-Ct_(PGD)_, ΔΔCt is ΔCt_(treatment)_- ΔCt_(control)_. Experiments were done in triplicate. Primer sets: PSMA forward: 5′-TCTGCTCGCGCCGAGATGTG-3′; PSMA reverse: 5′-ATTTTATAAACCACCCGAAG-3′; TMPRSS2/ERG forward: 5′-TAGGCGCGAGCTAAGCAGGAG-3′; TMPRSS2/ERG reverse: 5′-ACGCGGTCATCTGTGTCTTA-3′; PGD forward: 5′-AGACCATCTTCCAAGGCATTG-3′; PGD reverse: 5′-GTGGTATGCCTCACAGATCAG-3′.

### Luciferase reporter assay

VCaP cells were transfected with plasmid for PSM-Luc (contains PSMA promoter and enhancer) and Renilla-Luc luciferase reporter genes. Twenty-four hours post-transfection, cells were cultured in DMEM medium containing 9% charcoal-stripped fetal bovine serum (C-FBS, Hyclone) +1% normal FBS, and cultured for another 24 hours in the presence or absence of R1881, flutamide, or bicalutamide. Prior to harvest, LNCaP cells were co-transfected with TMPRSS2-ERG fusion transcript isoforms and PSM-Luc and Renilla-Luc. Twenty-four hours post-transfection, cells were cultured in phenol red-free RPMI 1640 (Invitrogen) containing 9% charcoal-stripped serum (CSS) +1% normal serum, and cultured for another 24 hours in the presence or absence of R1881. Cells were harvested, and firefly and *Renilla* luciferase activities were measured using the Dual-Luciferase Reporter Assay System according to the manufacturer's instruction (Promega).

### Chromatin Immunoprecipitation

Chromatin immunoprecipitation (ChIP) was performed by using a ChIP kit according to the manufacturer's instructions (ab500, Abcam Inc.). In brief, about 3×10^6^ VCaP cells were fixed in 37% formaldehyde before lysis. Chromsomal DNA was sheared by using a sonicator to an optimal DNA fragment size of 200–1000 bp. Anti-ERG (sc-354, Santa Cruz) antibody was used in the immunoprecipitation to pull down ERG protein and DNA complex. Rabbit IgG was used as negative control. After DNA purification, 0.5 ul each of input DNA, ERG-enriched, or rabbit IgG-enriched DNA were subjected to PCR analysis. A 192 bp fragment of PSMA promoter was amplified by the upstream 5′-TGCACGGCCTCTCTCACGGA-3′ and downstream 5′-GGCTATGTCTGGCTACTGTCTTA-3′ primers.

### Statistical analysis

For real-time PCR, the ΔCt values of treatment groups and vehicle control were compared using 2-sided paired t-tests which blocked on experimental replicates. For the luciferase assay, we analyzed data by using two sample t-test due to the pooled nature of the experiment. Significance was accepted when p<0.05.

## Results

### Inhibition of PSMA expression by the nonaromatizable androgen R1881 in VCaP cells

**Figure 1:**
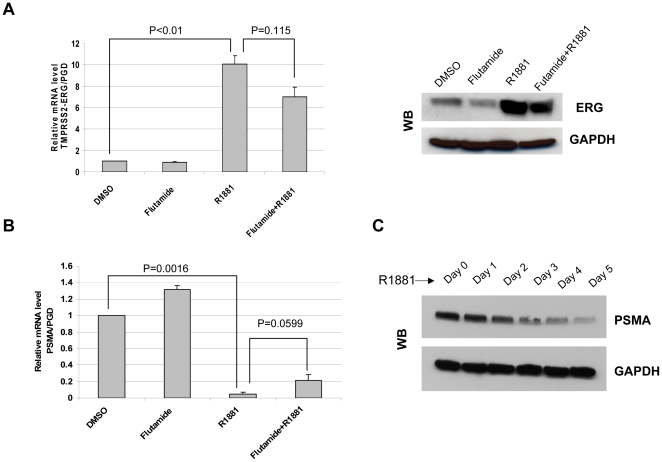
Inhibition of PSMA by androgen in VCaP cells. A, Expression levels of TMPRSS2-ERG in VCaP cells by real-time PCR, normalized to PGD mRNA level. ERG protein level was measured by western blot after treatment with 5 nM of R1881 or 10 µM of antagonist flutamide for 1 day. B, PSMA expression was detected by real-time PCR in VCaP cells, normalized to PGD. (Cells were treated the same as in Fig 1A). C, PSMA protein levels were checked by western blot in VCaP cells treated with 5 nM of R1881 for 0–5 days. For real-time PCR, cells were treated with vehicle or androgen antagonist flutamide for 2 hours prior to the treatment of the synthetic androgen R1881 for 24 hours. Experiments were done in triplicate.

Both VCaP and LNCaP cells were treated with vehicle or the androgen antagonist flutamide prior to treatment with the synthetic androgen R1881. After 24 hours treatment, TMPRSS2-ERG mRNA and protein levels were induced in VCaP cells by R1881, and the increased expression was attenuated by the addition of flutamide (Fig. 1A). PSMA mRNA levels dramatically decreased in VCaP cells after R1881 exposure for 24 hours, and treatment with flutamide partially restored PSMA mRNA level (Fig. 1B). After R1881 treatment for 3 days, PSMA protein levels were also decreased in VCaP cells (Fig. 1C). Since PSMA protein has a long half life of 55 hours [23], it took a longer time to decrease its protein level than to decrease mRNA level by R1881. Reports have shown PSMA is up-regulated by androgen ablation [12]; however, we found that the response of PSMA to androgen treatment was different in VCaP and LNCaP prostate cancer cell lines. R1881 significantly reduces PSMA in VCaP cells while it has a modest decrease in LNCaP cells. Interestingly, we found AR mRNA levels in response to its ligand were also different in these two cell lines. AR mRNA is dramatically reduced by R1881 in VCaP cells, while it has minimal change in LNCaP cells (data not shown). Our result is consistent with Makkonen's findings published recently [24]. VCaP cells harbor the *TMPRSS2-ERG* fusion and contain wild type AR, while LNCaP cells lack this fusion gene and have a mutated AR. Therefore, it is possible that the differential inhibition of PSMA by androgen treatment in VCaP cells and LNCaP cells is due to either the TMPRSS2-ERG fusion or the different type of AR. Moreover, AR expression level is higher in VCaP cells than in LNCaP cells, and the pattern for regulation of AR target gene expression by amplification of AR is also different in these two cell lines [24]. To clarify this point, we further determined PSMA expression following siRNA knock down of ERG in VCaP cells.

### ERG knockdown enhances PSMA expression in VCaP cells

**Figure 2:**
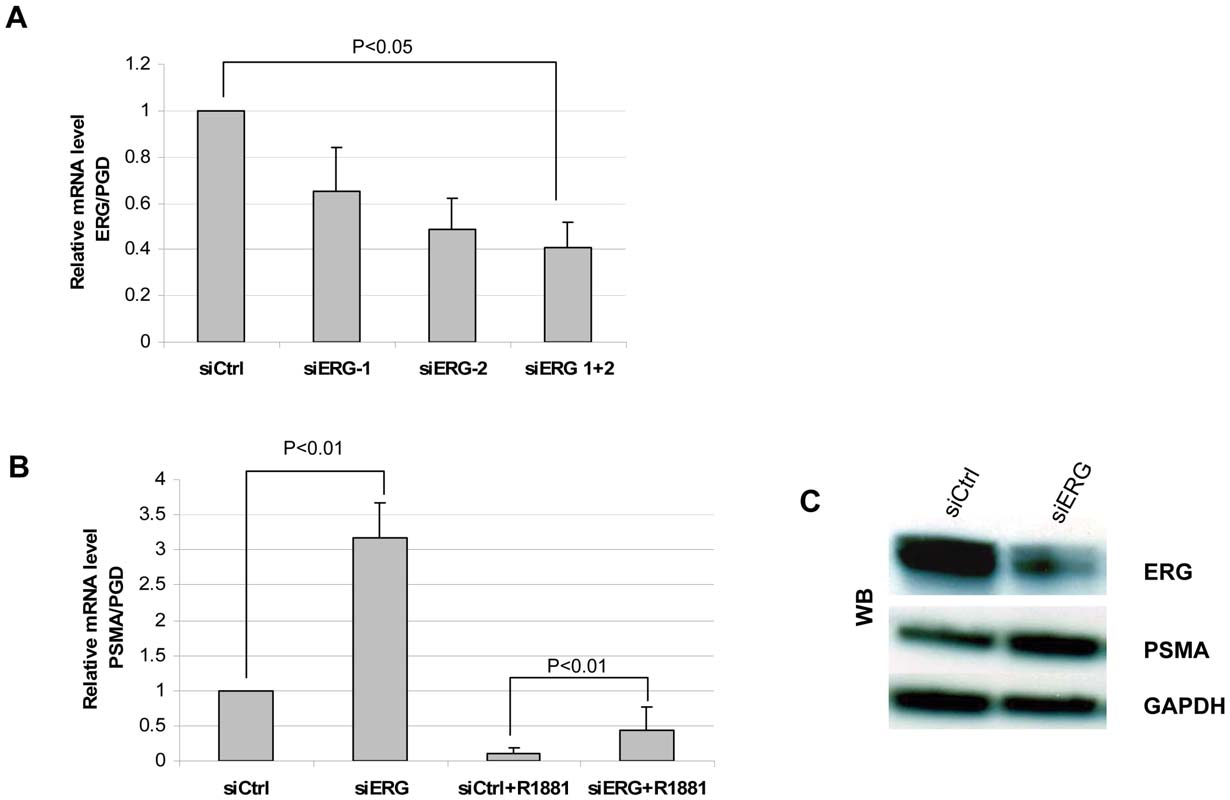
Knocking down ERG by siRNA enhances PSMA expression in VCaP cells. A, Cells were transfected with different ERG siRNA by oligofectamine for 48 hours. ERG expression levels were tested by quantitative real-time PCR, normalized to PGD mRNA level. B, Real-time PCR for PSMA expression in ERG knockdown VCaP cells. Cells were treated with or without R1881 for 24 hours after 48 hours treatment of siRNA. C, Western blot for ERG and PSMA expression in ERG knockdown VCaP cells. Cells were harvested 72 hours post siRNA transfection.

It is reported that the TMPRSS2-ERG fusion transcripts encode truncated ERG proteins at the amino terminus [19] and the vast majority of total ERG mRNA represents fusion gene transcripts. Therefore, by knocking down ERG, we can effectively evaluate the function of the TMPRSS2-ERG fusions. VCaP cells were transfected with siRNA for 3 days. Knocking down ERG was verified by real-time PCR. ERG mRNA level was reduced by 50% using specific ERG siRNA (Fig. 2A). Further knocking down of ERG was achieved by using combination of two siRNA siERG-1 and siERG-2 (Fig. 2A). So this siRNA combination was used in the following experiment. Partial silencing of ERG alone can enhance PSMA expression in VCaP cells (Fig. 2B, 2C). In addition, R1881-induced downregulation of PSMA expression was partially attenuated in ERG knockdown VCaP cells (Fig. 2B). These data indicate that either or both of the TMPRSS2-ERG fusion or wild type ERG can suppress PSMA expression in VCaP cells. To verify what type of ERG was involved in PSMA expression, next we checked their effects by overexpressing TMPRSS2-ERG fusion or wild type ERG in LNCaP cells.

### Overexpression of TMPRSS2-ERG fusions decreased PSMA expression in LNCaP cells

**Figure 3:**
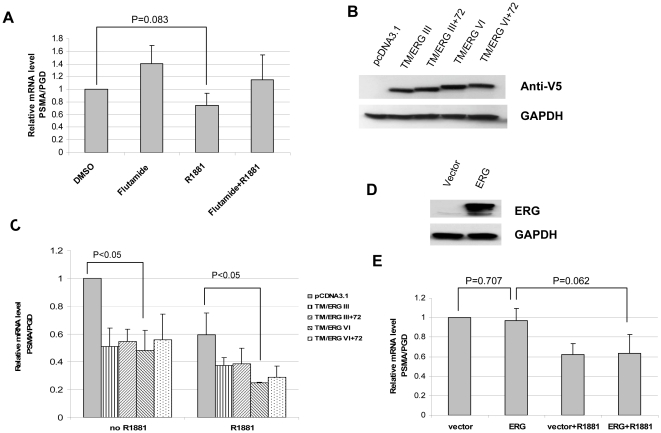
Overexpression of *TMPRSS2-ERG* fusions decreased PSMA expression in LNCaP cells. A, PSMA expression was detected by real-time PCR in LNCaP cells, normalized to PGD mRNA level. Cells were treated with vehicle DMSO or androgen antagonist flutamide for 2 hours prior to the treatment of the synthetic androgen R1881 for 24 hours. B, TMPRSS2-ERG fusion protein levels were checked by Western blot using anti-V5 antibody. LNCaP cells were transfected with fusion type III, III+72, VI, VI+72, or empty vector for 48 hours. C, Real-time PCR detected PSMA mRNA level in TMPRSS2-ERG fusion-transfected LNCaP cells. Cells were transfected with fusions or empty vector for 48 hours, then treated with or without R1881 for 24 hours. D, ERG expression level in ERG-transfected LNCaP cells by western blot. Cells were transfected with full length ERG for 48 hours. E, PSMA mRNA level in ERG overexpressing LNCaP cell. Cells were treated with or without R1881 for 24 hours post 48 hours of transfection. Experiments were done in triplicate.

TMPRSS2-ERG fusion isoforms have variable biological activities [18]. Of the fusion isoforms, the most common transcripts are type III (TMPRSS2 exon 1 fused to ERG exon 4) and type VI (TMPRSS2 exon 2 fused with ERG exon 4). Type III encodes a truncated ERG protein, while type VI encodes a true fusion protein [18]. Recently a 72-bp exon was found in some alternatively spliced fusion isoforms. The presence of the 72 bp exon has a significant biological function in cell proliferation [18]. Both of the type III+72 and type VI+72 isoforms can enhance cell proliferation. To further identify whether inhibition of PSMA is mediated by wild type ERG or by different TMPRSS2-ERG fusions, we transfected these ERG fusion constructs into LNCaP cells, which is ERG fusion-negative. The dosage of Lipofectamine 2000 used for transfection has minimal cytotoxicity on cell growth (data not shown). PSMA mRNA level had a modest decrease in LNCaP cells after R1881 exposure for 24 hours (Fig. 3A). When LNCaP cells were transfected with TMPRSS2-ERG fusion constructs, ERG fusion proteins were detected by using anti-V5 antibody (Fig. 3B). We found PSMA was downregulated, and its suppression was enhanced in the presence of R1881 (Fig. 3C). On the other hand, when LNCaP cells were transfected with normal ERG (Refseq: NM_004449.3), no change in PSMA expression was observed in this cell line (Fig. 3D, 3E). When treated with R1881, which can partially inhibit PSMA expression, the extent of inhibition was to the same extent both in the presence or absence of ERG (Fig. 3E). Given that the transfection efficiency in LNCaP cells is only around 50% by Lipofectamine (data not shown), and that we only found partial inhibition of PSMA in LNCaP cells by transient transfection of TMPRSS2-ERG fusions, more complete inhibition of PSMA expression would likely be achieved with a higher transfection efficiency. We did not see any different effect on PSMA inhibition among the different types of alternate TMPRSS2-ERG transcripts. Our data raised the possibility that inhibition of PSMA by androgen in VCaP cells may be mediated by truncated ERG or fusion proteins that are encoded by *TMPRSS2-ERG*, not by normal ERG protein.

### Inhibiton of PSMA luciferase activity by TMPRSS2-ERG

**Figure 4:**
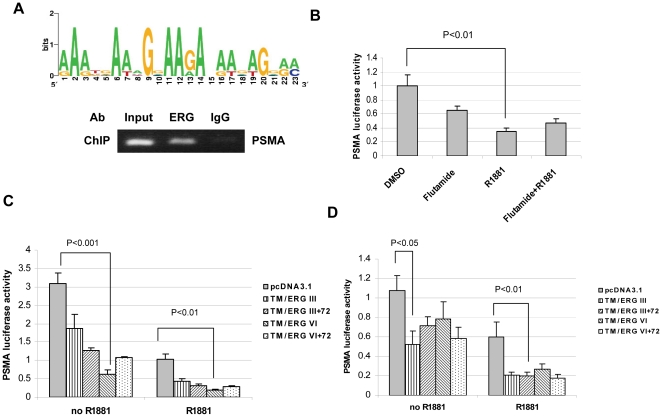
PSMA luciferase activity in VCaP or LNCaP cells. A, Graphic prediction of ETS transcription factor binding site on PSMA promoter and recruitment of ERG to PSMA promoter detected by ChIP assay. B, PSMA luciferase activity in VCaP cells. VCaP cells were transfected with PSM-Luc and Renilla-Luc luciferase reporter genes for 24 hours, then cells were treated with R1881 and androgen antagonist flutamide for another 24 hours. C and D, PSMA luciferase activity in LNCaP and C4-2 cells. LNCaP and C4-2 cells were co-transfected with PSM-Luc, Renilla-Luc, and TMPRSS2-ERG fusions, type III, III+72, VI, VI+72. Twenty-four hours post transfection, cells were cultured in the presence or absence of R1881 for another 24 hours.

According to MAT inspector software (http://www.genomatix.de/index.html), we found potential ETS transcription binding sites on the PSMA promoter and enhancer (data not shown). One of these binding sites on the PSMA promoter (nt 943-963) was highly conservative, according to computational prediction (http://genome.ucsc.edu/index.html; http://weblogo.berkeley.edu/) (Fig. 4A). We hypothesized that TMPRSS2-ERG can regulate PSMA at the transcription level. Therefore, we performed a ChIP assay and also transfected a luciferase reporter gene that contains the PSMA promoter and enhancer sequences into both VCaP and TMPRSS2-ERG-transfected LNCaP cells or its derivative cell line, C4-2. Recruitment of ERG to the *PSMA* promoter in VCaP cells was detected by ChIP assay (Fig. 4A). PSMA luciferase activity was inhibited in VCaP cells when treated with R1881 for 24 hours. Flutamide can reverse this inhibition (Fig. 4B). LNCaP and C4-2 cells that were cotransfected with TMPRSS2-ERG fusion isoforms and the PSMA luciferase reporter gene showed that different TMPRSS2-ERG isoforms alone can inhibit PSMA transcription activity, and the activity was further inhibited by R1881 treatment (Fig. 4C and 4D). These data confirm that TMPRSS2-ERG fusion can inhibit PSMA expression at the transcription level.

## Discussion

Increased PSMA expression has been correlated with a high Gleason score of disease and with tumor recurrence in prostate cancer. In this study, we found PSMA was downregulated by *TMPRSS2-ERG* fusion in VCaP prostate cancer cells. These are the first data to identify a mechanism for androgen signaling pathways involved in PSMA regulation. Recent reports indicated that the *TMPRSS2-ERG* fusion gene was present and expressed in pre-surgery androgen ablation patients [25]. In addition, ERG was found in circulating tumor cells from patients with castration-resistant prostate cancer [26]. Androgen ablation inhibits the expression of *TMPRSS2-ERG*. Moreover, patients with expression of the fusion gene had earlier prostate specific antigen (PSA) recurrence after radical prostatectomy [25]. Our findings demonstrate that, in VCaP cells, TMPRSS2-ERG fusions, but not wild type ERG, suppress PSMA expression. As there is an inverse relationship between PSMA expression and ERG fusions, it seems unlikely that studies claiming high expression of PSMA correlates to higher percentage and earlier failure of PSA would also be occurring in those the TMPRSS-ERG protein is expressed. Developing results from ongoing studies of prostate tissue microarrays demonstrated a negative correlation between PSMA expression and ERG expression in Gleason Score 7 prostate cancer (communication with Dr. Magi-Galluzzi Cristina from Cleveland Clinic), which is consistent with our *in vitro* findings.

Androgen signaling is important for prostate cancer growth and survival. Androgen exerts its biological functions through binding to the AR [27]. Overexpression of AR was found in castration-resistant prostate cancer [28], [29]. Investigators reported that high levels of AR in androgen deprivation therapy downregulated *TMPRSS2-ERG* fusion. The fusion gene can be up-regulated at a late time by reactivating AR in castration-resistant prostate cancer [30]. Also, ERG can disrupt AR signaling by inhibiting AR expression [31]. In our study, we found that PSMA could be a potential downstream target of *TMPRSS2-ERG* fusion in the AR signaling pathway. Since there is a correlation between AR and TMPRSS2-ERG expression, more work needs to be done to further address the role of AR in ERG fusion mediated PSMA expression.

We observed that PSMA regulation by androgen could be mediated by the *TMPRSS2-ERG* fusion. Knocking down mutant ERG can increase PSMA expression. This finding suggests that, in men with prostate cancers bearing the TMPRSS2-ERG fusion, a short course of androgen ablation might upregulate PSMA and facilitate therapeutic targeting and/or imaging based on PSMA.

The androgen receptor remains an important target. Androgen synthesis inhibitors and new more effective antiandrogens are demonstrating encouraging responses in castrate resistant disease. Still, resistance to these therapies will eventually develop, and other non-androgen based therapies such as linked toxin targeting may add to the arsenal of therapeutic agents available for treatment. Since PSMA is a promising target for prostate cancer therapy and imaging, further elucidation of the relationship between *TMPRSS2* gene fusions and PSMA could reveal novel pathways for enhancing targeted prostate cancer treatment.
